# The role of S100A14 in epithelial ovarian tumors

**DOI:** 10.18632/oncotarget.1947

**Published:** 2014-05-06

**Authors:** Hanbyoul Cho, Ha-Yeon Shin, Sunghoon Kim, Jane Seon-Young Kim, Joon-Yong Chung, Eun Joo Chung, Kyung-Hee Chun, Stephen M. Hewitt, Jae-Hoon Kim

**Affiliations:** ^1^ Department of Obstetrics and Gynecology, Gangnam Severance Hospital, Yonsei University College of Medicine, Seoul, Republic of Korea; ^2^ Department of Obstetrics and Gynecology, Severance Hospital, Yonsei University College of Medicine, Seoul, Republic of Korea; ^3^ Institute of Women's Life Medical Science, Yonsei University College of Medicine, Seoul, Republic of Korea; ^4^ Tissue Array Research Program & Applied Molecular Pathology Lab., Laboratory of Pathology, National Cancer Institute, National Institutes of Health, Bethesda, MD, USA; ^5^ Radiation Oncology Branch, Center for Cancer Research, National Cancer Institute, National Institutes of Health, Bethesda, MD, USA; ^6^ Department of Biochemistry and Molecular Biology, Yonsei University College of Medicine, Seoul, Republic of Korea

**Keywords:** Epithelial ovarian cancer, tumor marker, S100A14, shRNA

## Abstract

S100A14 is an EF-hand calcium-binding protein that has been reported to be involved in the progression of many malignancies. However, its role in ovarian cancer has not yet been clarified. In this study, we investigated the significance of S100A14 expression in epithelial ovarian cancers (EOCs) as well as it's mechanism of action. On both RNA and protein levels, S100A14 was overexpressed in transformed cells. Immunohistochemical staining demonstrated that S100A14 expression was associated with advanced stage (*P* < 0.001) and poor tumor grade (*P* < 0.001). Moreover, S100A14 overexpression was an independent prognostic factor for overall survival (HR = 4.53, *P* = 0.029). We also investigated S100A14's functional role by employing lentiviral-mediated overexpression and knockdown in EOC cells. S100A14 overexpression promoted cell proliferation, tumorigenesis, migration, and invasion, whereas S100A14 knockdown inhibited these properties. TOV112D cells that overexpressed S100A14 also exhibited greater tumor growth potential in xenografted mice. S100A14 promoted such a malignant phenotype in EOC cells through the PI3K/Akt pathway. Taken together, our data indicate that S100A14 has a crucial role in EOC progression, and its overexpression is associated with poor prognosis. Further study of S100A14's molecular mechanisms may lead to the development of a novel therapeutic target for ovarian cancer.

## INTRODUCTION

Ovarian cancer is the leading cause of death from gynecological malignancies in developed countries [1]. Despite their clinical importance, little is known about the early stages of development for this neoplasm, largely owing to the absence of adequate animal models, the clinical inaccessibility of human ovaries, and the unfortunate paucity of symptoms until higher stages. The most common form of ovarian cancer is epithelial ovarian cancer (EOC). EOCs are believed to originate from normal ovarian surface epithelium or from its derivatives, which include the crypts and inclusion cysts on the epithelial surface [2]. A series of recent evidence also suggest that ovarian high-grade serous carcinoma (HGSC) originates from the fimbrial portion of the fallopian tube, which has been termed “serous tubal intraepithelial carcinoma” (STIC) [3-6]. Understanding the molecular basis of EOC should significantly refine the diagnosis and management of these tumors and eventually lead to the development of more specific and more effective treatment modalities.

S100 proteins, a large subgroup of the EF-hand protein family, are small calcium-binding proteins that can function as both intracellular and extracellular signaling molecules [7]. They exert a broad range of functions by modulating their subcellular localization and by interacting with specific target proteins responsible for the regulation of inflammation, cell growth, cell motility, cell survival, and apoptosis [7-9]. Altered expression of a large number of S100 proteins has been reported in various human malignancies [10]. In addition, some S100 proteins, namely S100A2, S100A4, and S100P, have been implicated in tumor invasion and metastasis [11-13].

S100A14 has recently been identified as an S100 calcium-binding protein with unknown biological function [14]. The S100A14 gene was originally cloned and characterized in human lung cancer cell line, but was found to be differentially expressed in a variety of cell types. It was also reported to be upregulated in several tumor types, including ovarian, lung, breast, and uterine cancer, but downregulated in others, such as kidney, colon, rectal, and esophageal cancer [14]. S100A14 has also been shown to play vital roles in bladder tumorigenesis and tumor progression [15]. S100A14 can also regulate oral squamous cell carcinoma cell invasion by modulating the expression of matrix metalloproteinase (MMP) 1 and MMP9 [16]. However, the role of S100A14 in ovarian cancer development and its underlying molecular mechanisms remain unclear. We have previously identified S100A14 to be upregulated in EOC by microarray [17] and have confirmed this by real-time PCR. In this study, we further investigate the clinical significance and functional role of S100A14 in EOC.

## RESULTS

### High S100A14 expression correlates with poor prognosis in EOC

**Figure 1:**
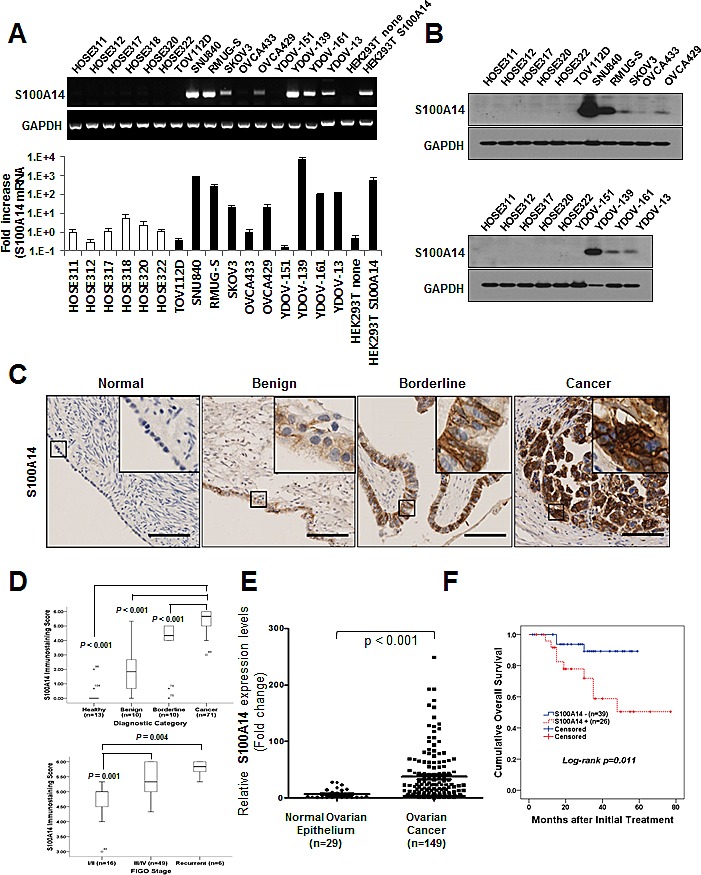
S100A14 is highly expressed in human ovarian cancer cells and tissue specimens, and its expression correlates with tumor stage and outcome of disease A. *S100A14* mRNA levels were assessed using RT-PCR (upper panel) and real-time PCR (lower panel) in human ovarian surface epithelial (HOSE) cells and ovarian cancer cells. Expression of GAPDH was included as an internal loading control. Kruskal-Wallis analysis of variance and post hoc Dunn method revealed statistically significant differences between EOC and HOSE cell lines (*P* = 0.019). Each value is expressed as a mean of triplicate samples. The reference cell line, HOSE 311, was considered to have a value of 1. B. S100A14 protein levels were analyzed using immunoblot. GAPDH was included as an internal loading control. C. Representative immunohistochemical staining for S100A14 in formalin-fixed, paraffin-embedded EOC tissues. Bars: 100 μm. D. IHC staining scores of S100A14 in EOC samples were significantly higher than those of healthy controls, benign ovarian tumors, and borderline ovarian tumors (*P* < 0.001). E. When we combined seven microarray data sets from GEO database, S100A14 mRNA expression was significantly increased in ovarian cancer (n = 149) when compared to normal ovarian surface epithelium (n = 29) (*P* < 0.001). F. Kaplan-Meier plots for patients with epithelial ovarian cancer were stratified according to S100A14 expression.

We previously observed that the transcription level of *S100A14* was upregulated 7.85-fold in three EOC cell lines by microarray analysis [17]. To eliminate the possibility that S100A14 gene expression only occurred in newly established EOC cell lines in *in vitro* culture, we performed validation studies using PCR and immunohistochemistry (IHC) in various EOC cell lines and tissues. Reverse transcriptase-PCR (RT-PCR) and real-time PCR revealed thatS100A14 mRNA levels were abundantly expressed in ovarian cancer cell lines, except TOV112D, OVCA433, and YDOV-151, whereas S100A14 expression was almost undetectable in HOSE cell lines (Fig. 1A). Lysates from HEK293T cells transfected with pCDH/S100A14 plasmids were loaded as a positive control. Expression of S100A14 at the protein level was confirmed by immunoblot (Fig. 1B). S100A14 was highly expressed in SNU840, RMUG-S, and YDOV-139 cell lines, but could not be detected in HOSE cell lines (Fig. 1B). These observations suggest that S100A14 expression primarily occurs in fully transformed cells.

To determine whether S100A14 overexpression is linked to clinicopathological features of EOC, we performed IHC analysis of S100A14 in 104 ovarian tissue specimens. Most immunoreactivity was observed in the cytoplasm of malignant cells (Fig. 1C). Scoring results from IHC analyses are summarized in Supplementary Table 2. EOC tissues had higher S100A14 expression levels than in borderline, benign, or normal tissues (*P* < 0.001). Furthermore, S100A14 immunoreactivity significantly correlated with features also associated with poor prognosis, including tumor stage (*P* < 0.001), histologic subtype (*P* = 0.004), and tumor grade (*P* < 0.001). Specifically, advanced stage including recurrence, serous histologic subtype, and poor differentiation correspond to higher IHC scores (Fig. 1D, Supplementary Table 1). To further analyze S100A14 expression in ovarian cancer, we combined seven microarray data sets (GSE55510, GSE55512, GSE27651, GSE14001, GSE14407, GSE28724, and GSE51373) downloaded from the GEO database according to the literature [14]. Overall, 149 ovarian cancers from genome-wide gene expression data were available. As expected, we found that S100A14 mRNA expression was significantly higher in ovarian cancer (n = 149) than in normal ovarian surface epithelium (n = 29) (*P* < 0.001).

We next examined the relationship between S100A14 expression and outcome. Clinicopathological and outcome information was available for 65 EOC patients. Tumor samples in six cases were obtained at recurrence and excluded from the survival analysis. The follow-up period of EOC ranged from 5 to 77 months with a mean of 30.8 months. Kaplan-Meier plots demonstrated that patients with advanced stage (III/IV) and patients whose tumors were S100A14+ (IHC score of 6) displayed significantly worse overall survival (*P* = 0.029 and *P* = 0.011, respectively) (Fig. 1E, Supplementary Fig. 1A-1C). A Cox multivariate proportional hazards analysis showed that advanced stage (hazard ratio [HR] = 3.25, *P* = 0.038 and HR = 4.31, *P* = 0.024, respectively) and S100A14+ status (HR = 3.10, *P* = 0.017 and HR = 4.53, *P* = 0.029, respectively) were independent prognostic factors of disease-free and overall survival (Table 1).

**Table 1:** Univariate and multivariate analyses of the associations between prognostic variables and disease-free (DFS) and overall survival (OS) in EOC patients

	DFS hazard ratio (95% CI), P value		OS hazard ratio (95% CI), P value	
	Univariate	Multivariate	Univariate	Multivariate
FIGO stage (III/IV)	4.78 [1.86-12.23], P=0.001	3.25 [1.07-9.92], P=0.038	4.84 [1.62-14.48], P=0.005	4.31 [1.20-15.44], P=0.024
Tumor grade (poor)	2.49 [1.11-5.59], P=0.026	1.03 [0.43-2.46], P=0.938	NS	NA
Cell type (Serous)	4.49 [1.33-15.11], P=0.015	1.40 [0.36-6.41], P=0.665	NS	NA
CA125+†	NS	NA	NS	NA
S100A14+‡	6.74 [2.82-16.07], P=0.001	3.10 [1.22-7.89], P=0.017	6.42 [1.40-29.34], P=0.016	4.53 [1.16-17.69], P=0.029

CI, confidence interval; NS, not significant; NA, not applicable, CA125+† (> 35 U ml−1), S100A14+‡ (IHC score of > 5).

### Lentiviral-mediated overexpression and knockdown of S100A14 reveal that high S100A14 expression increases cell proliferation and colony formation in EOC cells

**Figure 2:**
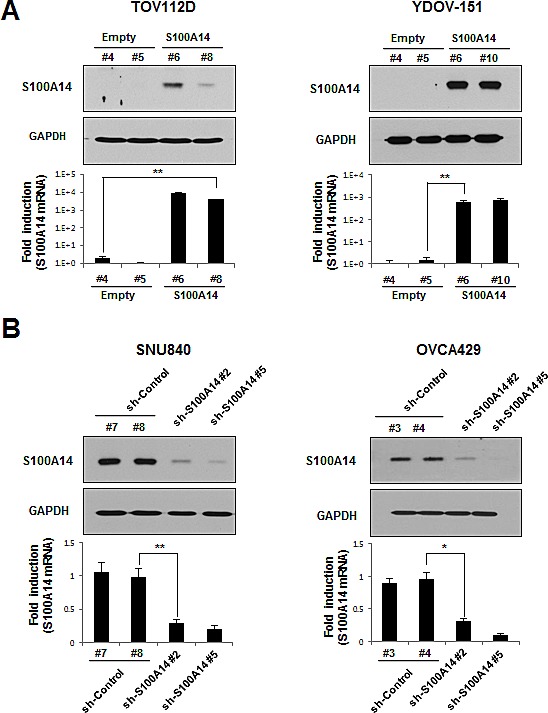
Ectopic S100A14 expression and knockdown in lentivirus-mediated stable cells A and B. Whole cell lysates and total RNA pool were collected from TOV112D (A, left : transfected with empty clone #4, #5 or *S100A14* clone #6, #8), YDOV-151 (A, right : transfected with empty clone #4, #5 or *S100A14* clone #6, #10). SNU840 (B, left: transfected with empty clone #7, #8 or *S100A14* shRNA sequence #2, #5), or OVCA429 (B, right: transfected with empty clone #3, #4 or *S100A14* shRNA sequence #2, #5) stable cell lines. All shRNA sequences were different from each other. Expression of S100A14 protein was analyzed by immunoblot (upper panels), and mRNA level was measured by real-time PCR (lower panels). GAPDH was included as an internal loading control. An asterisk (*) indicates a *p*-value < 0.05, and a double asterisk (**), a *p*-value < 0.01.

Given that the biological function of S100A14 in EOC is largely unknown, we set out to investigate the potential role of S100A14 in the development of a malignant phenotype in EOC cells by modulating intracellular S100A14 expression. We first established stable S100A14-overexpressed and -knocked down EOC cell lines by antibiotic selection of a pool of lentivirus-infected cells. For our S100A14 overexpression experiments, we used TOV112D and YDOV-151 cell lines, which have low levels of endogenous S100A14. Conversely, we used SNU840 and OVCA429 cell lines that have high levels of endogenous S100A14 for our knockdown experiments. Strong S100A14 immunoreactivity indicated excellent enrichment of S100A14 in TOV112D (*S100A14 #6, #8*) and YDOV-151 (*S100A14 #6, #10*) cells when compared to empty vector-transfected cells. Real-time PCR results were similar to those obtained from immunoblots, wherein higher S100A14 mRNA expression was shown in exogenous S100A14-expressing cells (Fig. 2A). In contrast, protein levels of S100A14 decreased by short-hairpin S100A14 RNAi in SNU840 (*sh-S100A14 #2, #5*) and OVCA429 (*sh-S100A14 #2, #5*) cells (Fig. 2B). Ultimately, the two most efficiently overexpressing or knocked down cell cultures for each cell type were selected to further investigate phenotypic changes arising from S100A14 overexpression or downregulation.

**Figure 3:**
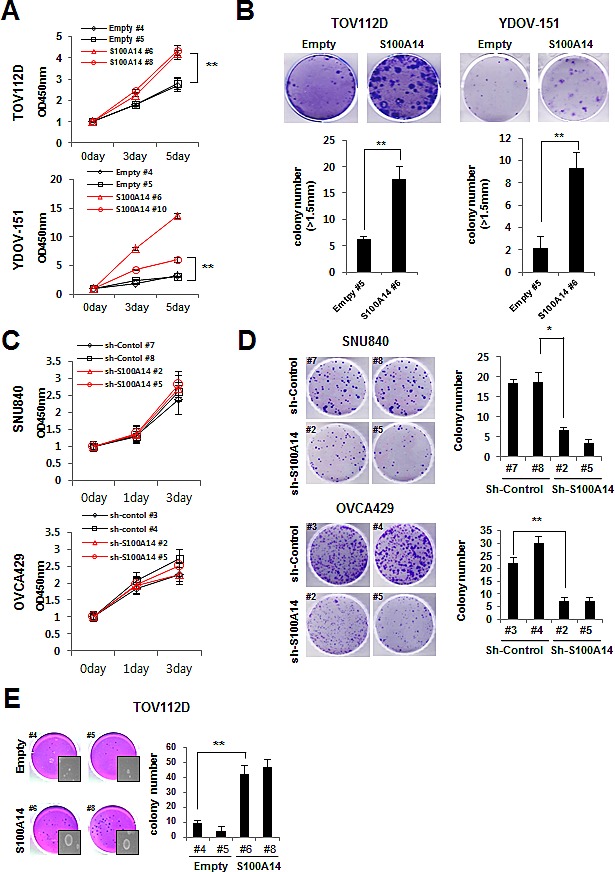
S100A14 increases cell proliferation and clonogenicity A. Cell proliferation was determined by WST-1 assay. Cell proliferation curves for TOV112D and YDOV-151 cells at various indicated times. Error bars represent mean ± SD of triplicate experiments. B. A clonogenic assay was performed on TOV112D and YDOV-151 cells for 3 weeks. Left panel shows representative images; and right panel shows quantification of colonies. Error bars represent mean ± SD of triplicate experiments. C. Cell proliferation was determined by WST-1 assay. Cell proliferation curves for SNU840 and OVCA429 cells at various indicated times. Error bars represent mean ± SD of triplicate experiments. D. A clonogenic assay was performed on SNU840 and OVCA429 cells for 3 weeks. Left panel shows representative images; right panel shows quantification of colonies. Error bars represent mean ± SD of triplicate experiments. E. Soft-agar colony formation assay was performed using TOV112D cells. Left panel shows a representative picture; right panel shows quantification of colonies. Error bars represent mean ± SD of triplicate experiments. An asterisk (*) indicates a *p*-value < 0.05, and a double asterisk (**), a *p*-value < 0.01.

We analyzed the effect of S100A14 overexpression in TOV112D and YDOV-151 using two assays to investigate short-term proliferative impact (WST-1 assay) and long-term proliferative ability (colony formation assay). In all S100A14-overexpressing cell lines, rates of cell growth were significantly higher than in empty vector-expressing controls (Fig. 3A). Similar results were also observed in colony formation assays (Fig. 3B). When S100A14 expression was repressed in SNU840 or OVCA429 cells, cell growth was not significantly altered by the WST-1 assay (Fig. 3C). However, knockdown of S100A14 significantly decreased colony formation in SNU840 and OVCA429 cells when compared to the shRNA-control group (Fig. 3D). We next performed a soft agar colony assay to assess S100A14's role in tumorigenesis and found that the number of colonies formed from S100A14-transfected TOV112D cells was significantly higher than those resulting from empty vector-expressing controls (Fig. 3E).

### Lentiviral-mediated overexpression or knockdown of S100A14 reveals that high S100A14 expression increase cell motility and invasion in EOC cells

**Figure 4:**
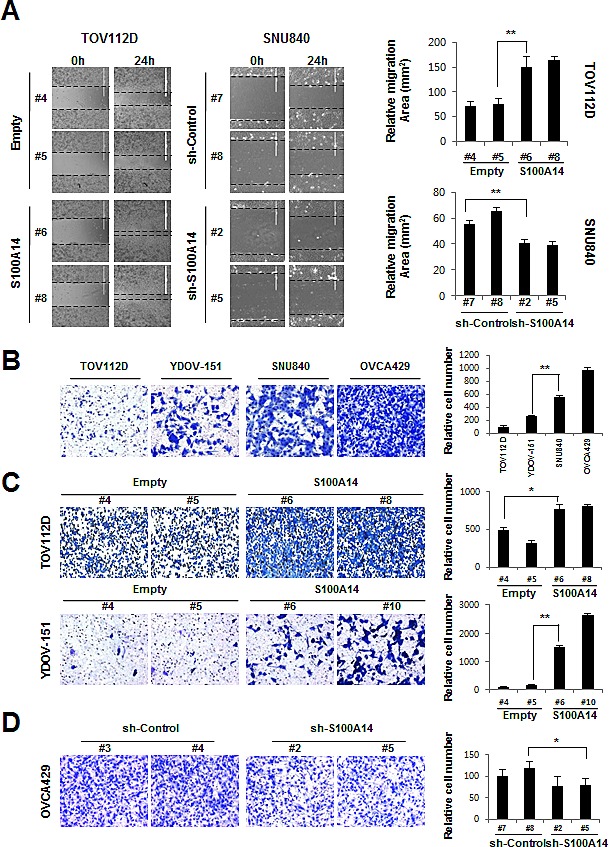
S100A14 promotes cell migration and invasion A. Cell migration analyses of TOV112D and SNU 840 cells by wound healing assay. Left panel: Representative images of migrations assays. Right panel: Quantitative results of migration experiments. Results are presented as relative migration area. B-D. Cell invasion analysis of untreated EOC cells (B), TOV112D (C), YDOV-151 (C) and OVCA429 (D) cells using Matrigel invasion assay. Left panel: Representative figures of cell invasion. Right panel: Quantitative results of invasion experiments. Results are presented as relative number of invading cells. Cells were counted in 4 randomly selected fields. An asterisk (*) indicates a *p*-value < 0.05, and a double asterisk (**), a *p*-value < 0.01.

To investigate whether S100A14 plays a role in migration in EOC cells, we conducted a wound-healing assay for cell migration. Overexpression of S100A14 resulted in increased motility of TOV112D cells when compared to empty vector-expressing cells (Fig. 4A). In contrast, shRNA-mediated S100A14 knockdown inhibited cell motility in SNU840 cells (Fig. 4A). To evaluate whether S100A14 could also enhance invasion in EOC cells, a Matrigel invasion assay was performed. SNU840 and OVCA429, both of which have higher levels of endogenous S100A14, demonstrate significantly higher invasive potential than TOV112D and YDOV-151, which both express endogenous S100A14 at much lower levels (Fig. 4B). To further confirm the possible role of S100A14 in cell invasion, we evaluated the invasive potential in TOV112D, YDOV-151, and OVCA429. S100A14 overexpression in TOV112D (*S100A14#8*) and YDOV-151 (*S100A14 #6, #10*) resulted in increased invasion when compared to empty vector-expressing controls (Fig. 4C). In contrast, S100A14 knockdown in OVCA429 (*sh-S100A14 #2, #5*) cells led to a decrease in invasion capability (Fig. 4D). To explore the molecular mechanism behind S100A14's promotion of cell migration and invasion, we characterized the expression of matrix metalloproteinase genes, MMP1, MMP2, and MMP9, by RT-PCR (Supplementary Fig. 2A). Interestingly, among the MMPs detected, expression of MMP1 and MMP9 was dramatically upregulated in S100A14-overexpressed TOV112D cells. Real-time PCR analysis further confirmed the dramatic increase of MMP1 and MMP9 expression (Supplementary Fig. 2B). However, no significant difference in p53 expression was observed between S100A14-overexpressed cells and empty vector-transfected cells (Supplementary Fig. 2C-D). Taken together, these results demonstrated that S100A14 plays an important role in cell motility and invasion.

### S100A14 overexpression in TOV112D cells increases tumor growth in nude mice xenografts

**Figure 5:**
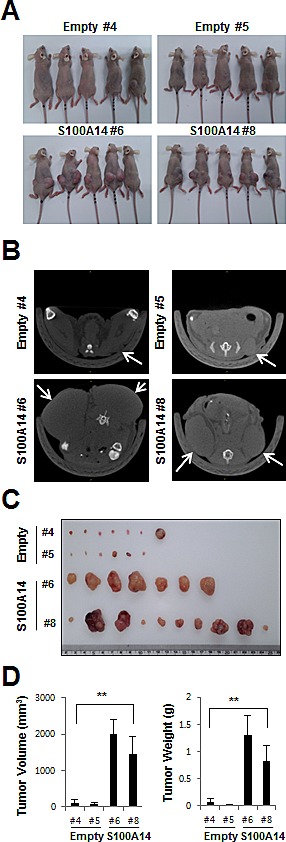
S100A14 overexpression increases in vivo xenograft tumor growth A. For the xenograft, a total of 1.5x10^6^ cells were injected subcutaneously into the left and right flank, respectively. B. Representative microCT images of mice seven weeks after injection. C. Gross images of tumor masses from representative mice from each group. D. S100A14 overexpression increased tumor volume and weight of xenografted TOV112D cells in BALB/c-nu mice. Mean tumor volume and weight for each group was calculated at seven weeks after injection. Error bars were calculated as standard error of means, and two-way ANOVA was used for statistical analyses (*n* = 5 mice/group). A double asterisk (**) indicates a *p*-value < 0.01.

To explore whether S100A14 can affect tumor growth *in vivo*, we inoculated TOV112D (*S100A14* #6, #8, Empty #4, #5) as xenografts into nude mice (Fig. 5A). Tumor volume and weight were measured. Mean tumor volumes (1998.5±397.0 and 1442.4±491.7 mm^3^, respectively) at day 49 in mice receiving S100A14-overexpressed TOV112D (*S100A14* #6, #8) cells were significantly larger than those (114.8±97.8 and 86.3±64.7 mm^3^, respectively) in mice receiving empty vector-expressing cells (Empty #4, #5) (*P* < 0.001) (Fig. 5B-D). Tumor weight correlated with tumor volume, as determined by calipers (*P* < 0.001; r^2^ = 0.935) (Fig. 5D). These data indicated that S100A14 can promote tumor growth *in vivo* and further support our initial hypothesis that S100A14 plays a functional role in the malignant transformation of EOC.

### S100A14 promotes a malignant phenotype in EOC cells through the PI3K/Akt signaling pathway

**Figure 6:**
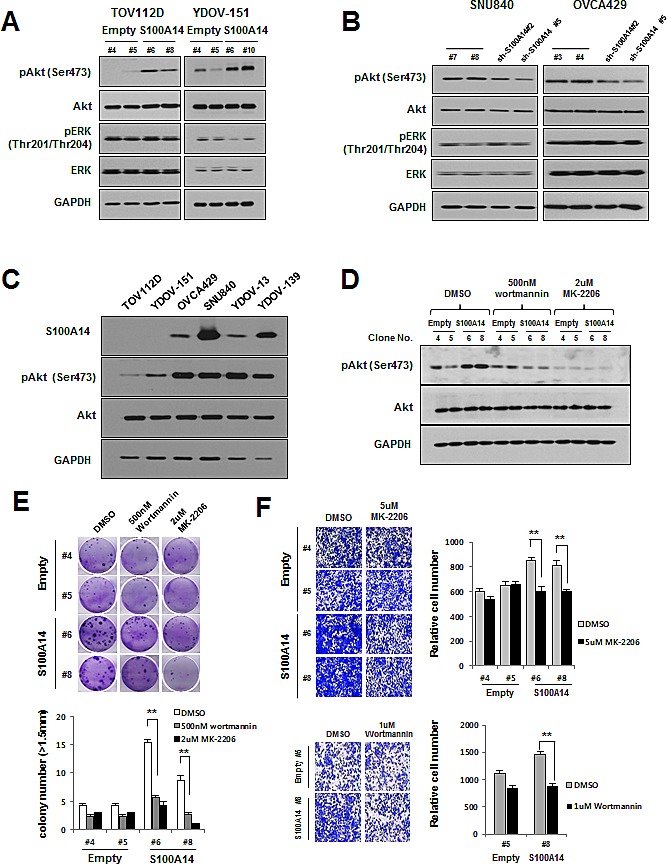
S100A14 controls oncogenic phenotypes of EOC cells through PI3K/Akt pathway A and B. Expression of pAkt (ser478), Akt, pERK (thr201/204), and ERK was assessed by immunoblot in TOV112D and YDOV-151 cells (A) and SNU840 and OVCA429 cells (B). C. Protein levels of S100A14, pAkt (ser478), and Akt were determined by immunoblot in non-transfected EOC cells. GAPDH was included as an internal loading control. D. Immunoblot analysis of pAkt inhibition in TOV112D cells treated with wortmannin, MK-2206, or control (DMSO) for eight hours. E. A clonogenic assay was performed on TOV112D cells for three weeks in the presence of wortmannin, MK-2206, or control (DMSO). Left panel shows representative images, and right panel shows quantification of colonies. Error bars represent mean ± SD of triplicate experiments. F. Cell invasion analysis of TOV112D cells with wortmannin, MK-2206, or control (DMSO). All drugs were applied to the upper chamber. Left panel: Representative figures of cell invasion. Right panel: Quantitative results of invasion experiments. Results are presented as relative number of invading cells. Cells were counted in six randomly selected fields. A double asterisk (**) indicates a *p*-value < 0.01.

To determine the mechanism by which S100A14 promotes a malignant phenotype in ovarian cells, we assessed the status of important signaling cascades controlled by Akt and Erk in S100A14-overexpressed and S100A14-shRNA-knockdown cells. S100A14 overexpression in TOV112D (*S100A14* #6, #8) and YDOV-151 (*S100A14* #6, #10) cells led to increased pAkt expression (Fig. 6A), while S100A14 knockdown in SNU840 (sh-*S100A14* #2, #5) and OVCA429 (sh-*S100A14* #2, #5) cells led to decreased pAkt expression (Fig. 6B). There was no detectable change, however, in either pErk or Erk expression after S100A14 transduction. To confirm the relationship between S100A14 and pAkt, we prepared immunoblots in a series of non-transfected EOC cells. TOV112D and YDOV-151 cells, which lack endogenous S100A14 expression, showed lower levels of pAkt expression compared to cells with higher endogenous S100A14 expression (Fig. 6C). These data suggest that S100A14 could potentially modulate the activity of Akt.

To define the role of Akt in S100A14-modulated cell proliferation and invasion, we treated TOV112D (*S100A14* #6, #8) cells with either a PI3K (Akt upstream kinase) inhibitor, wortmannin, or an Akt inhibitor, MK-2206, and evaluated their effects on cell proliferation and invasion. First, we confirmed the specificity of these inhibitors by immunoblot (Fig. 6D), wherein increased levels of pAkt in S100A14-overexpressed cells were significantly decreased by both wortmannin and MK-2206. Next, we observed that wortmannin and MK-2206 both significantly decreased S100A14-enhanced cell proliferation and invasion via colony formation assay and invasion assay (Fig. 6E and 6F). Taken together, these data demonstrate that S100A14 controls the invasive potential and proliferative ability of cells through regulation of the PI3K/Akt pathway.

## DISCUSSION

In addition to identifying novel diagnostic markers for ovarian cancer, further understanding of the molecular mechanisms underlying the progression and metastasis of ovarian cancer is needed to develop more effective therapeutic treatments. Among various genes and proteins previously identified to be expressed or modified in ovarian cancer[17], S100A14 attracted research interest due to its marked and consistent overexpression in all six EOC cell lines and its reported role in cell growth and survival in other cancers [18-20]. In the present study, we investigated the functional role and clinical significance of S100A14 expression in ovarian cancer using EOC cell lines and mouse xenograft models.

It has recently been proposed that ovarian cancer can be divided into two main categories, type I and type II tumors, based on their distinct pathogenesis[21]. Type I tumors include well-differentiated serous, mucinous, endometrioid, malignant Brenner, and clear cell tumors that generally exhibit slow, indolent growth with *Ras* and/or *Raf* mutations. In contrast, type II tumors include moderately or poorly differentiated serous, undifferentiated, and malignant mixed mesodermal tumors with p53 mutation. Type II tumors are highly aggressive and present at an advanced stage, where currently available therapies are seldom curative[22]. When we consider the fact that type II tumors are more aggressive than type I tumors and constitute approximately 75% of ovarian cancer [23], it is reasonable to focus on type II tumors when identifying potential biomarkers in the future. We therefore performed subgroup analyses of IHC results in type I and type II tumors to compare the molecular subtypes of ovarian cancer. S100A14 protein expression was greater in type II tumors (IHC score = 5.42, n = 52) when compared to type I tumors (IHC score = 4.85, n = 19) (*P* < 0.001). Although the difference was small in our study, subsequent analysis using more samples may demonstrate a significant difference in S100A14 expression between type I and type II tumors. Notably, S100A14+ status and advanced tumor stage were independent prognostic factors for both disease-free and overall survival on multivariate analysis (Table 1). Taken together, these results suggest that S100A14 may play an important role in the pathogenesis of EOC. However, it should be noted that our relatively small clinical sample size is a limitation of this study and that the small sample size and mixture of different histological subtypes may weaken the strength of our clinical analysis. We therefore plan to incorporate larger numbers of specific histological subgroups to generalize our findings in a future study.

Increasing evidence suggests a critical role for the S100 family in cell growth, invasion, and cancer metastasis. We therefore investigated the potential roles of S100A14 in EOC based on our prior analysis of clinical specimens. S100A14 overexpression resulted in significant increases in cell proliferation and clonogenicity. Conversely, S100A14 knockdown led to the suppression of these phenotypes (Fig. 3). Importantly, S100A14 expression positively correlated with cell migration and invasion in EOC cell *in vitro* cultures (Fig. 4) and *in vivo* xenograft-bearing mice (Fig. 5). Another limitation of our study should be noted, however. Only subcutaneous xenograft models were used to investigate EOC cell behavior *in vivo*. Cancer cells implanted subcutaneously allow for rapid and quantitative tumor formation, being more suitable for studies that require continuous measurement of the tumor. In contrast, intraperitoneal or orthotopic xenograft models are inherently difficult to quantitatively monitor tumor growth, but can provide a more relevant tumor microenvironment. Despite this limitation, our findings suggest that S100A14 is one of the critical proteins contributing to EOC carcinogenesis and progression.

Interestingly, there was a discrepancy in the level of S100A14 expression (Fig. 2A) with the gain of phenotype, such as cell growth (Fig. 3A), colony formation (Fig. 3E), migration (Fig. 4A), and invasion (Fig. 4C) in S100A14-transfected TOV112D and YDOV-151 stable cell lines. There may be many reasons for the observed discrepancy, such as the characteristics of the cell line itself (e.g., TOV112D originates from endometrioid EOC and YDOV-151 is from mucinous EOC), cell shape, and cellular localization of S100A14. It is well known that the function of protein could be controlled by cell shape or its subcellular localization [24,25]. Thus, it will be very useful to examine cell morphology and/or subcellular localization of S100A14 in TOV112D and YDOV-151 stable cell lines in future studies.

It is well known that some S100 proteins regulate cell invasion and metastasis by modulating expression and activity of MMPs [11,16,33]. In particular, Chen et al. reported that S100A14 promotes cell motility and invasion by regulating the expression and function of MMP2 in a p53-dependent manner in esophageal squamous cell carcinoma [25]. In the present study, MMP1 and MMP9 expression was enhanced in S100A14-overexpressed TOV112D cells (Supplementary Fig. 2A-2B). However, S100A14 overexpression did not increase the expression of p53 in TOV112D cells (Supplementary Fig. 2C-D). We believe that this is mainly because of TOV112D's p53R175 mutant status (http://www.atcc.org/products/all/CRL-11731.aspx#characteristics). In contrast to our study, Sapkota et al. demonstrated that S100A14 overexpression was associated with suppression of MMP9 gelatinolytic activity in CaLH3 cells[16]. Thus, the function of S100A14 can be different depending on the anatomical origin of these malignant cells.

Finally, we investigated whether Akt or Erk signaling pathway is compromised upon S100A14 overexpression or knockdown. There was an obvious relationship between increased levels of phosphorylated Akt and overexpression of S100A14 in TOV112D and YDOV-151 cells (Fig. 6A). Conversely, S100A14 knockdown decreased phosphorylated Akt in SNU840 and OVCA429 cells (Fig. 6B). However, there was no detectable change in either expression or activation of Erk1/2 after S100A14 modulation (Fig. 6A-B). Inhibition of PI3K/Akt signaling with wortmannin or MK-2206 resulted in significant decreases in phosphorylated Akt (Fig. 6D), cell proliferation (Fig. 6E), and cell invasion (Fig. 6F) in TOV112D or YDOV-151 cells. Thus, S100A14 may contribute to the malignant phenotype of EOC cells through activated PI3K/Akt signaling.

In summary, we demonstrate that S100A14 expression was lowest in normal ovarian epithelium, slightly increased in benign and borderline ovarian tumors, and highest in EOC. Enhanced S100A14 expression correlated positively with clinicopathological parameters in both *in vitro* and *in vivo* ovarian cancer systems, lending support for the use of S100A14 to determine clinicopathological stage and/or prognosis in ovarian cancer. In addition, S100A14 could be a potential therapeutic target, as the present study demonstrated the mechanistic role of S100A14 in promoting cell motility and invasion through the PI3K/Akt pathway.

## METHODS

### Cell lines and reagents

Five EOC cell lines (YDOV-13, YDOV-151, YDOV-139, YDOV-161, and YDOV-13) and 6 human ovarian surface epithelial (HOSE) cells, which were established and characterized in our laboratory, were cultured as described previously [28,29]. SNU-840 was purchased from Korean Cell Line Bank (KCLB, Seoul, Korea). RMUG-S was purchased from Health Science Research Resources Bank (HSRRB, Osaka, Japan). SKOV3, TOV112D, OVCA429, and OVCA433 cell lines were purchased from American Type Culture Collection (ATCC, Manassas, VA). All purchased cell lines were maintained as recommended. AKT inhibitor MK-2206 was purchased from Sellect (Houston, TX), and PI3 kinase inhibitor wortmannin was purchased from Sigma Aldrich (St. Louis, MO). The stock solutions of 10 mM MK-2206 and 1 mM wortmannin were dissolved in dimethyl sulfoxide (DMSO).

### Patients and tumor specimens

Tumor samples from 71 EOCs (54 serous, 6 mucinous, 4 endometrioid, 2 transitional cell, 2 mixed, 2 clear cell, and 1 Brenner tumor), 10 borderline ovarian tumors (6 serous and 4 mucinous), 10 mucinous cystadenomas, and 13 normal ovary specimens were included in this study and were provided by the Korea Gynecologic Cancer Bank through Bio & Medical Technology Development Program of the Ministry of Education, Science and Technology, Korea. Tumor staging was performed according to the International Federation of Gynecology and Obstetrics (FIGO) classification. All FIGO Stage I/II ovarian cancer patients underwent pelvic and para-aortic lymph node dissection according to the National Comprehensive Cancer Network (NCCN) clinical practice guidelines. Medical records were reviewed to collect data including age, surgical procedure, survival time, and survival status. Response to therapy was assessed according to Response Evaluation Criteria in Solid Tumors (RECIST; version 1.0) by spiral computed tomography [30]. Data for tumor grades and cell types were obtained by reviewing pathology reports. All biological samples were obtained with appropriate informed consent from participants according to institutional review board (IRB) guidelines.

### Immunohistochemistry

Paraffin tissue sections were deparaffinized in two changes of xylene, rehydrated in graded ethanol, and treated for 30 min with 3% H_2_O_2_ solution in methanol to block endogenous peroxidase. After blocking in 10% goat serum in TBS for 30 minutes, sections were incubated with rabbit polyclonal anti-Human S100A14 antibody (Proteintech Group, Inc., Chicago, IL) diluted to 1:100 for 1 hr at room temperature, followed by detection using Dako LSAB+ (Dako, Glostrup, Denmark). The reaction product was developed with DAB (3,3'-diaminobenzidine) chromogen solution (Dako). Sections were counterstained with hematoxylin and mounted in Faramount aqueous mounting medium (Dako). Appropriate negative and positive controls were concurrently performed. Representative photomicrographs were recorded using a digital camera (Nikon, Tokyo, Japan). Negative controls were processed by omitting the primary antibody. Human pancreas tissue was used as a positive control for S100A14 immunoreactivity.

Staining for S100A14 was scored as positive when tumor or epithelial cells showed cytoplasmic immunoreactivity. S100A14 staining results were scored based on staining intensity (0 = negative, 1 = weak, 2 = moderate, 3 = strong) and the percentage of positive cells (0 = 0%, 1 = 1 – 25%, 2 = 26 – 50%, 3 = 51 – 100% positive cells), as described previously [31]. For the immunostaining score, the intensity and positivity scores were added, resulting in a value between 0 and 6. The overall score for each patient was further simplified by dichotomizing as either negative (overall score of ≤ 5) or positive (score of 6). Slides were scored in the absence of any clinical data, and the final immunostaining score was the average score of three expert pathologists.

### Real-time PCR

Total RNA was extracted using an RNeasy Mini Kit (Qiagen, Valencia, CA). cDNA was generated using a SuperScript ™ III First–Strand Synthesis System (Invitrogen, Carlsbad, CA). Real-time PCR was performed to quantify messenger RNA expressions using SYBR^®^ Green PCR Master Mix (Applied Biosystems, Foster City, CA) and the ABI PRISM^®^ 7300 real-time PCR system (Applied Biosystems). A housekeeping gene, glyceraldehyde-3-phosphate dehydrogenase (GAPDH), was used to normalize the quantity of cDNA used in the PCR reaction. Each assay was performed in triplicate and expressed as the mean ± SD. The primers used for the PCR analysis were as follows: for *S100A14* forward 5'-GTG TCG GTC AGC CAA CGC AGA-3', *S100A14* reverse 5'-TGC TGG GTG ACC AGG TCC CGT-3', *MMP1* forward 5'-CTG CTG CTG CTG TTC TGG GGT-3', *MMP1* reverse 5'-CCA CTG GGC CAC TAT TTC TCC GCT-3', *MMP2* forward 5'- GAT ACC CCT TTG ACG GTA AGG A -3', *MMP2* reverse 5'CCT TCT CCC AAG GTC CAT AGC -3', *MMP9* forward 5'- AGA CGG GTA TCC CTT CGA CG -3', *MMP9* reverse 5'- AAA CCG AGT TGG AAC CAC GAC -3', *GAPDH* forward 5'- GAA GGT GAA GGT CGG AGT -3', *GAPDH* reverse 5'- GAA GAT GGT GAT GGG ATT TC -3'. The primer for TP53 was purchased from Bioneer (Daejeon, Korea; Cat.# P188330). The comparative C_T_ method was used to calculate relative quantification of gene expression as described previously [32].

### Transfection and generation of stable cell lines

To generate pCDH/*S100A14*, cDNA encoding human *S100A14* was amplified from pOTB7 *S100A14* cDNA clone (MHS1011-60727, Open Biosystems) using the primer set 5'-TTC TAG AGC CAC CAT GGG ACA GTG TCG GTC AG-3' (forward) and 5'-TTG CGG CCG CTC AGT GCC CCC GGA CAG-3' (reverse). The amplified cDNA was cloned into *Not* I/*Xba* I restriction sites of the pCDH-Promoter-MCS-EF1 Lentivector (System Biosciences, Mountain View, CA). Stable cell lines expressing S100A14 in TOV112D and YDOV-151 were generated using the pPACKH1 Lentivector Packaging Kit (System Biosciences), and selection of S100A14-transduced cells was accomplished using 2.0 μg/mL puromycin (Invitrogen).

For the generation of sh-S100A14 stable cell lines, pLKO.1 *S100A14* shRNA libraries were purchased from Open Biosystems (Waltham, MA). Of five lentiviral constructs tested, two with the best knockdown efficiency were used for the experiments presented here, using the human *S100A14* sequence ATCACTGAATTCCTGAGCATC for shRNA #2 and TGGTGAAAGTTCTTGATGAGG for shRNA #5. The non-target shRNA control vector (SHC002) was purchased from Sigma (Sigma Aldrich). Stable cell lines expressing sh-S100A14 were generated using the viral packaging plasmids composed of pCMV delta and pMDG. Virus particles were collected after 48 hours and 72 hours post-transfection. SNU840 and OVCA 429 cells were transduced, and positively transduced cells were selected with puromycin (2 μg/mL).

### Immunoblotting

Whole cell extraction was conducted using PRO-PRE Protein Extraction Solution (Intron Biotechnology, Seongnam, Korea). Equal amounts (20 μg) of each sample were separated on 8-15% SDS-PAGE and transferred to nitrocellulose membranes. The membranes were blocked with 5% nonfat dry milk in TBST (50 mM Tris, 150 mM NaCl, 0.1% Tween-20, pH 7.5) for 1 hr at room temperature, washed with TBST, and subsequently incubated with primary antibodies: anti-S100A14 (Proteintech, Chicago, IL), anti-phospho-Akt (ser473), anti-Akt, anti-phospho-Erk1/2, anti-Erk1/2 (Cell Signaling, Danvers, MA), anti-TP53 (Santa Cruz Biotechnology, Santa Cruz, CA) and anti-GAPDH (Santa Cruz Biotechnology). Primary antibodies against each protein were detected by secondary antibodies conjugated with horseradish peroxidase (GE Healthcare, Munich, Germany). Specific bands for each protein were detected on AGFA X-ray film (Agfa Health Care, Mortsel, Belgium) using the SuperSignal Chemiluminescence kit (Thermo Scientific, Rockford, IL).

### Cell growth and clonogenicity

Cell proliferation was measured by WST-1 assay (DaeilLab, Seoul, Korea). In brief, cells were seeded at 3×10^3^ viable cells/well onto 96-well microtiter plates in a final volume of 100 μL/well. Cells were incubated with WST-1 at 37°C for 2 hours and optical density (OD) values at 450 nm were recorded at days 0, 3, and 5 using a 96-well microplate reader (Bio-Rad Laboratories, Inc., Hercules, CA). The experiment was performed in triplicate.

In order to examine clonogenicity, cells were seeded into 60-mm dishes (0.02-0.1×10^4^ cells/well) and cultured for 3 weeks. Colonies formed in each well were fixed with H_2_O containing 10% methanol and 10% acetic acid, stained with 0.5% crystal violet, and then counted visually. A three-dimensional clonogenic growth assay was performed using a soft agar culture system. Briefly, cells were mixed in 0.3% agar containing 10% FBS and layered on top of the base layer of 0.6% agar in a 60-mm dish (3×10^3^ cells/dish). After a four-week incubation in a humidified CO_2_ incubator, colonies were counted visually after staining with 0.5% crystal violet. Each cell group was cultured in triplicate, and colonies greater than 1.5 mm in diameter were scored visually.

### Cell migration and invasion assays

Cell migration was assessed by monolayer wound healing assay [33]. Briefly, cells were seeded into 12-well tissue culture dishes and allowed to grow to 90% confluency in complete medium. Cell monolayers were wounded with a plastic pipette tip (1 mm), and washed four times with complete medium to remove cell debris. After 24 hours of culture, phase contrast images were captured of the gap in the monolayers (EVOS®FL Cell Imaging System, Life Technologies, Carlsbad, CA). A cell invasion assay was performed in an invasion chamber (Neuro Probe 48-Well Micro Chemotaxis Chamber, Neuro Probe, Inc., Gaithersburg, MD) according to the manufacturer's instruction. In brief, 5×10^4^ cells were resuspended in serum-free medium (56 μL) and plated onto the upper chamber coated with Matrigel (BD Transduction Lab, San Jose, CA). The lower chamber was filled with medium containing 0.1-10% FBS (27 μL). After 24 hours of incubation, cells that had migrated through the membrane were stained with the Differential Quik Stain Kit (Triangle Biomedical Sciences, Inc., Durham, NC). Invading cells in six randomly selected fields were counted using Microscope Axio Imager.M2 (Carl Zeiss, Thornwood, NY, Magnification x200). Each experiment was repeated three times.

### Animal studies

All animal procedures were performed under a protocol approved by the Institutional Animal Care and Use Committee of Gangnam Severance Hospital. A mouse ovarian cancer xenograft model was established using female BALB/C nude mice aged 4-5 weeks (OrientBio Inc., Sungnam, Gyunggi, Korea). TOV112D cells (1.5×10^6^ cells/inoculation) were resuspended in 100 μL of a Matrigel (BD Biosciences, QC) and PBS mixture (1:1) and inoculated subcutaneously in both left and right flanks of each mouse. Seven weeks after inoculation, tumor-bearing mice were subjected to microCT imaging (NFR Polaris-G90 Micro CT scanner, Nano Focus Ray, Jeonju, Chunbuk, Korea). Briefly, mice were anesthetized using isoflurane/O_2_ (1.5-5% v/v) gas and scanned with the following parameter settings: 360 scan angle, X-ray voltage 65 kV, X-ray current 115 μA, X-ray spot size 9 μm, binning 1, and exposure time 40 ms. After microCT imaging, tumors were harvested from each mouse, weighted, and fixed in 10% neutral buffered formalin for histological assays. Tumor volumes were calculated using the following formula: tumor volume (mm^3^) = (*L × W^2^*)/2, where L is the length and W is width [34]. Data is represented as means ± standard errors.

### Statistical analysis

Normality of distribution was assessed using the Shapiro-Wilk test. Because the distribution was not normal, univariate comparisons for quantitative variables between normal and cancerous specimens were made using non-parametric statistics (Kruskal–Wallis and Mann–Whitney U) where appropriate. Results with two-tailed *p*-values less than 0.05 were considered statistically significant. Statistical analyses were performed using SPSS software version 18.0 (SPSS Inc., Chicago, IL).

## SUPPLEMENTARY FIGURES AND TABLES


