# Supplementary material for: The role of S100A14 in epithelial ovarian tumors
Source: Oncotarget. 2014 May 6;5(11):3482–96. doi: 10.18632/oncotarget.1947 (PMC4116497; doi:10.18632/oncotarget.1947)
Supplement: Supplementary file 1 [file oncotarget-05-3482-s001.pdf]

The role of S100A14 in epithelial ovarian tumors

Supplementary Material

Supplementary Table 1: S100A14 immunohistochemical staining score in EOC.

	No. of patients	Scores		*P* value
		Geometric mean (95% CI)	Range	
**Diagnostic category**				**< 0.001**
Healthy	13	0.20 (-0.13-0.54)	0.0-2.0	
Benign	10	1.90 (0.70-3.10)	0.0-5.3	
Borderline	10	3.76 (2.44-5.09)	0.0-5.0	
Cancer	71	5.37 (5.21-5.52)	3.0-6.0	
**FIGO stage**				**< 0.001**
I/II	16	4.72 (4.40-5.05)	3.0-5.3	
III/IV	49	5.51 (5.36-5.67)	4.3-6.0	
Recurrent	6	5.89 (5.71-6.06)	5.6-6.0	
**Histologic subtype**				**0.004**
Serous	54	5.49 (5.32-5.66)	3.0-6.0	
Non-serous	17	4.98 (4.71-5.24)	4.0-6.0	
**Tumor grade**				**< 0.001**
Borderline	10	3.76 (2.44-5.09)	0.0-5.0	
Well	8	4.70 (3.94-5.47)	3.0-6.0	
Moderate	33	5.30 (5.11-5.49)	4.0-6.0	
Poor	30	5.62 (5.41-5.82)	4.0-6.0	

CI, confidence interval; FIGO, International Federation of Gynecology and Obstetrics.

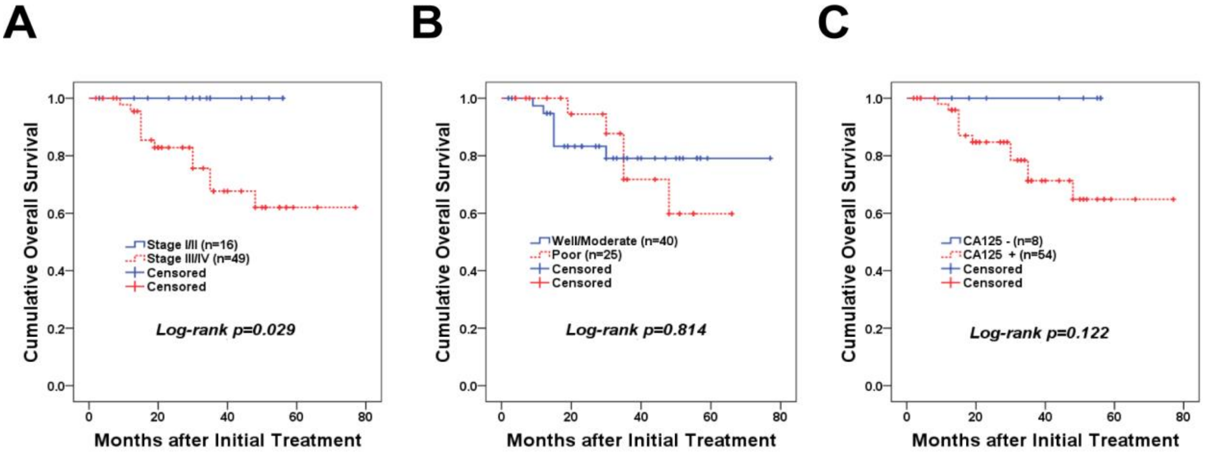

Supplementary Figure 1: Patient survival analyses. A-C. Kaplan-Meier plots for patients with epithelial ovarian cancer were stratified according to FIGO stage (A), tumor grade (B), or CA125 expression (C).

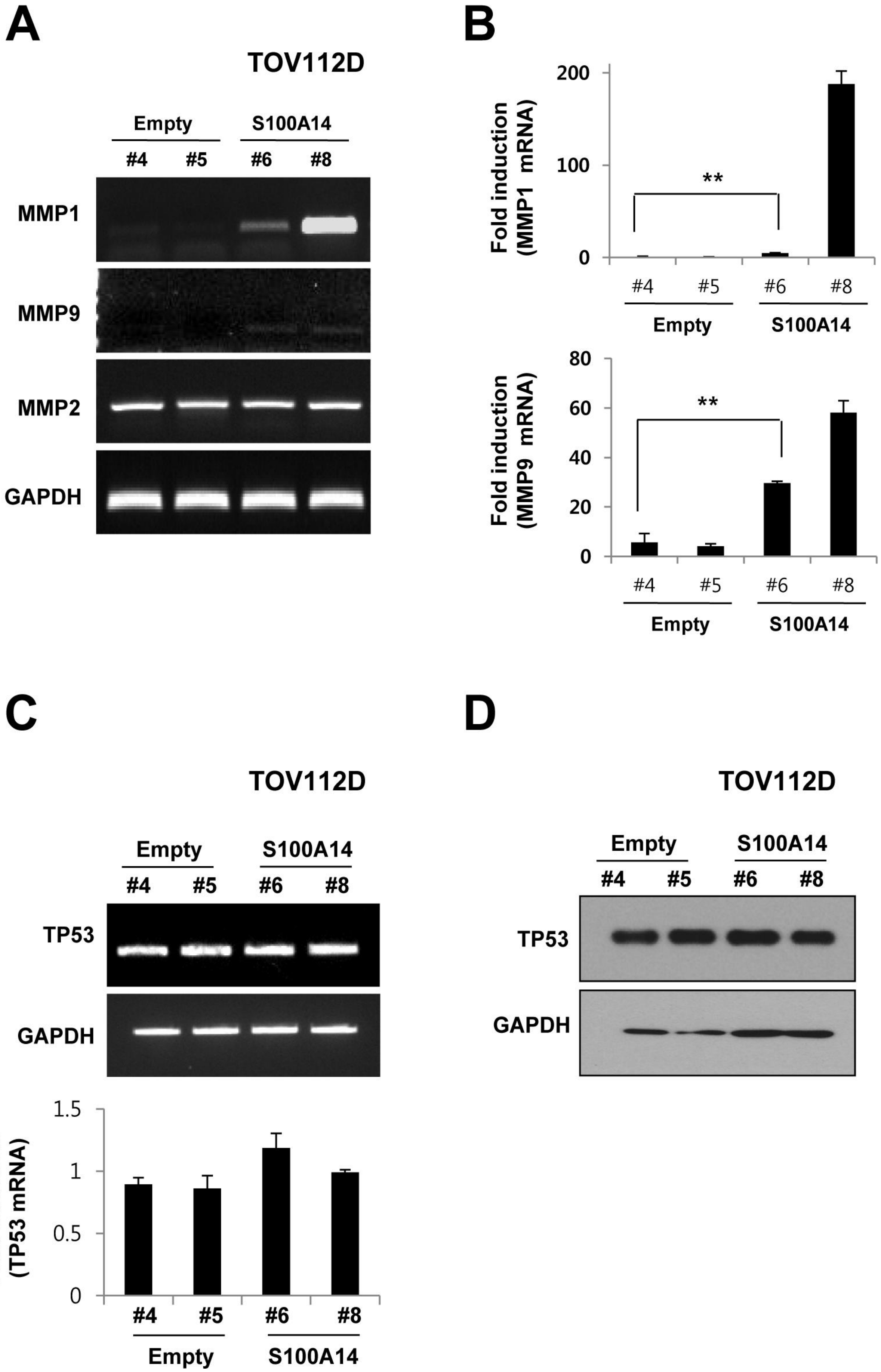
A
TOV112D
Empty
S100A14
#4
#5
#6
#8
MMP1
MMP9
MMP2
GAPDH
B
Fold induction (MMP1 mRNA)
200
100
0
**
#4
#5
#6
#8
Empty
S100A14
Fold induction (MMP9 mRNA)
80
60
40
20
0
**
#4
#5
#6
#8
Empty
S100A14
C
TOV112D
Empty
S100A14
#4
#5
#6
#8
TP53
GAPDH
Fold induction (TP53 mRNA)
1.5
1
0.5
0
#4
#5
#6
#8
Empty
S100A14
D
TOV112D
Empty
S100A14
#4
#5
#6
#8
TP53
GAPDH

Supplementary Figure 2: Expression of MMP1, MMP9, and MMP2. A and B. Matrix metalloproteinase (MMP) mRNA levels were assessed using RT-PCR (A) and real-time PCR (B) in TOV112D stable cells. C. TP53 mRNA levels were assessed using RT-PCR (upper panel) and real-time PCR (lower panel) in TOV112D cells. D. TP53 protein levels were analyzed using immunoblot. Expression of GAPDH was included as an internal loading control. Graphs show mean ± S.D. A double asterisk (**) indicates a *p*-value < 0.01.
